# Suicide in pediatrics: epidemiology, risk factors, warning signs and the role of the pediatrician in detecting them

**DOI:** 10.1186/s13052-015-0153-3

**Published:** 2015-07-07

**Authors:** Dario Dilillo, Silvia Mauri, Cecilia Mantegazza, Valentina Fabiano, Chiara Mameli, Gian Vincenzo Zuccotti

**Affiliations:** Department of Pediatrics, Ospedale dei Bambini V. Buzzi, University of Milan, Milan, Italy; Department of Pediatrics, Luigi Sacco Hospital, University of Milan, Milan, Italy

**Keywords:** Suicide, Risk factors, Protective factors, Warning signs, Antidepressive drugs

## Abstract

Epidemiological data suggests suicide is uncommon in childhood but becomes an extremely serious issue among adolescents.

Several risk factors have been identified and include the presence of psychiatric illness, a previous suicide attempt, family factors, substance abuse, sexual and physical abuse, disorders in gender identity or bullying. Pediatricians have a primary role in searching for these risk factors, recognizing them and acting synergistically with other specialists to prevent and treat suicidal behavior.

Pediatricians should also be able to identify the “warning signs” for suicide since their presence implies a need for immediate action, as attempted suicide may occur in a few hours or days.

The use of antidepressant drugs and its association with suicidal risk in pediatric age is another topic of ongoing debate. Food and Drug Administration has recently introduced the so-called “black box” on antidepressants’ packages with the aim of gaining attention to the possible risk of suicide among adolescents who are treated with antidepressants, with a warning that the risk of suicide is higher when starting a therapy or while adjusting its dosage.

## Introduction

Suicide is uncommon in children before the age of puberty; subsequently, its frequency tends to increase during adolescence. The pediatrician is involved in either identification or management of young people with suicidal behaviors. On the one hand, the pediatrician should be able to identify the presence of “warning signs” for attempted suicide/suicide in children and/or adolescents. Specifically, the American Academy of Pediatrics (AAP) recommends that a screening for suicide among adolescents should be carried out during visits performed for acute diseases as well as during routine check-ups [1]. On the other hand, the pediatrician should also provide patients with diagnostic and therapeutic support, collaborating with other professionals in order to create a team capable of managing all their different needs.

## Review

### Epidemiology

Most of the epidemiological data on suicide in pediatric age comes from American studies.

Prevalence of attempted suicide among adolescents has changed over the years: whereas a reduction was reported in the period from 2001 to 2008 (8.8 % - 7.8 %), on the contrary, an increase was subsequently observed in the 2009-2011 two-year period (6.3 % -7.8 %). On average, it is estimated that approximately 2 million adolescents are attempting suicide every year, 700.000 subjects in this age group request healthcare after an attempted suicide, 2.000 adolescents die [2]. Recent data from the Youth Risk Behavior Surveillance [3] shows that in 2011, 15.8 % of students between the ages of 14 and 18 years have considered suicide, 7.8 % have attempted suicide and 2.4 % have necessitated medical care afterwards. It is also estimated that while the attempted suicide is twice as frequent among females compared to males, death by suicide is more frequent among males than in females (9.4/100.000 versus 2.7/100.000). In addition, although the prevalence of death by suicide among adolescents remains low (0.006 %), suicide represents the third leading cause of death among subjects aged between 13 and 19 years, and the second leading cause of death among those aged between 10 and 24 years [4]. In Italy, suicide in the youngest represents one of the leading cause of death among people between the ages of 15 and 24; in 2007, 154 boys between the ages of 14 and 24 died because of suicide [5]. There are no specific Italian databases for the detection of attempted suicide; approximately, it is estimated that the ratio between attempted suicide and suicide in the youngest is 200:1. A recent italian study investigated the trends in the prevalence of suicide from January 2011 to December 2013 by sex and age; fifty-five suicide deaths among young people aged less than 18 years of age were identified, suicide rates were higher among Italians than foreign children/adolescents (8.16 times higher); the most frequent known cause of suicide was conflict in a romantic relationship, followed by bullying [6]. Epidemiologic data regarding suicide in pre-adolescents are scarce. Pelkonen et al. [7] reported a rate of suicide of 0.5 per 100.00 for females and of 0.9 per 100.00 for males amongst 5-14-year-olds. Dervic et al. [8] estimated a mean rate of suicide in children and young adolescents up to 14 years of age of approximately 0.6/100.00 worldwide, with a male-female ratio of 2:1. Childhood affective and disruptive disorders and abuse were the most often reported psychiatric risk factors. A recent French retrospective study [9] has analyzed the data from 48 subjects aged less than 13 years, who were hospitalized from 2008 to 2011 after attempted suicide in a department of child psychiatry. Attempted suicide was more frequent among the female subjects compared to males (F:M = 26:22). Age-specific differences were also reported: subjects who attempted suicide aged more than 12 years were predominantly females, whereas children attempting suicide under 12 years of age were predominantly males. Patients younger than 11 years had used more violent means to attempt suicide.

## The role of Pediatrician

Prevention of suicide is an extremely challenging issue for the healthcare providers. Some literature data reports that, in adulthood, the victims of suicide had often visited their doctor before attempting suicide [10]. Considering the increased incidence of suicide in adolescence, the pediatrician plays a vital role in assessing the level of risk by clinical judgment, by reviewing the risk and protective factors and by a direct verification of thoughts and acts of suicide. Moreover, the pediatrician plays a fundamental role in the field of education [1]. A recent American study [11] evaluating perception and ideas on suicide in 66 adolescents and 30 parents, showed that both categories recognized it as one important issue, but not a matter of significance to them. In addition, both adolescents and their parents recognized factors including mental illness, substance and alcohol abuse, as well as social difficulties, as common risk factors for suicide. However, parents also considered that transient phases of alcohol and substances abuse may be considered normal in adolescence. Both groups expressed the need to be informed and educated on the topic of suicide. It is evident that the pediatrician role is more complex than simply performing a screening for suicide: he/she should encourage families to address such issues as well as being involved in educational programs.

Even if there are no specific tests to identify subjects susceptible to suicide, risk factors must be investigated by the pediatrician and taken into consideration [12] (Table 1). Risk factors include:

**Table 1:** Risk factors for suicide among adolescents

Demographic	Clinical	Family and environmental	Mental State
- Male sex	- Psychiatric illness (major depression, bipolar disorder, conduct disorder)	- Particularly stressful events	- Thoughts on suicide
- Older age	- Recent discharge from a psychiatric hospital	- Availability of lethal means	- Abuse of alcohol or illegal substances
- Non heterosexual	- Previous attempted suicide	- Lack of social support	- State of anxiety, agitation, being without hope
	- Family history of depression or suicide	- Contact with subjects with suicidal behaviors	- Impulsiveness
	- Physical/sexual abuse	- Loss of a parent (death/divorce)	- Difficulty in troubleshooting
	- History of trauma in childhood	- Difficult relationship with parents	
	- Sleep disorders	- Perception of excessive control/poor care by parents	
	- Organic pathologies that determine functional limitations	- Bullying	
	- Personality disorders		
	- Low self-esteem		
	- Low compliance to therapy		

Adapted from Gordon M, Melvin G. Risk assessment and initial management of suicidal adolescents. Aust Fam Physician. 2014;43(6):367-72

- Psychiatric illness. Literature data shows that approximately 90 % of adolescents who commit suicide are suffering from a psychiatric disorder (in particular humor disorder), and more than 60 % of young people are depressed at the time of death [13]. A meta-analysis conducted in 2006 on 60.000 adolescents aged between 13 and 18 years old, estimated a prevalence of more than 5.6 % of subjects with depression (females > males) [14]. Moreover, some authors suggest that up to 20 % of adolescents have a depressive episode during the course of their life [15]. The Diagnostic and Statistical Manual of Mental Disorders (DSMV) [16] shows that children compared to adolescents have a greater difficulty in representing their internal states and they show depression through behavioral and somatic symptoms (abdominal pain, constipation, headache) and through irritable mood; adolescents instead show symptoms that are more similar to those experienced by adults such as depressed mood, loss of interest in daily activities, weight loss/gain and insomnia/hypersomnia (Table 2) [1]. In addition, some signs of depression can be noticed by teachers at school and must be investigated by the attending physician (Table 3). The process of screening for depression in pediatric age and adolescence is highly controversial: the disease can cause adverse effects both in the short term life of a child/adolescent and in the long term future life as an adult. In 2005 a Canadian Task Force stated that there is enough evidence to support the benefit of routine health screening for depression in these age groups [17]. However, in 2009 a US Task Force [18] argued that adolescents, but not children, should be screened for depression in primary care centers, when accurate diagnosis, psychotherapy and follow-ups are ensured. Nowadays the problem still remains open. Although the screening for depression is viewed as the only alternative to a complete indifference to the problem, many authors argue that investing resources in therapeutic programs rather than in screening would be preferable. In addition, very little is known about the possible positive effects of a screening program, while there is a fear that an increase in diagnosis without well-defined treatment guidelines could lead to a significant increase in prescription of antidepressant drugs without adequate programs of follow-up [19].

**Table 2:** Symptoms and signs of depression in the pediatric age

- Irritability and/or depressed mood
- Loss of interest in everyday activities
- Psychomotor agitation and/or loss of energy
- Low concentration and indecision
- Insomnia and/or hypersomnia
- Loss and/or increase in body weight
- Loss of hopes and sense of powerlessness
- Feelings of guilt and low self-esteem
- Chronic somatic symptoms, which do not respond to common therapies (headache, addominalgia, constipation)
- Thoughts of death and/or suicide

**Table 3:** Signs of depression at school

- Frequent absences
- Excessive delays
- Low concentration
- Difficulty in performing the tasks assigned
- Crying in class
- Difficulty in adapting to changes
- Poor participation in school activities
- Difficulty of interaction with companions
- Provocative attitude
- Restlessness and nervousness
- Isolation and muteness
- Previous attempted suicide. Literature data shows that a prior history of suicidal or parasuicidal behavior (attempts of suicide, past exposure to suicidal risk without a declared suicidal intention) represents an important risk factor for suicide. Self-injurious behaviors, defined as attitudes to cause direct and deliberate harm to oneself (eg non- suicidal self-injury, suicidal behavior and suicide) [20], are of health concern. Data from literature estimates that between 13 % and 29 % of adolescents engage in non-suicidal self-injury (self-cutting, burning, biting without lethal intent). Instead, suicidal behaviors (suicide attempt, sucide) refer to direct self-injurious behaviors with the intent to end one’s life such as hanging/strangulation, jumping from heights and severe cutting. Non suicidal self injury and suicidal behavior differ in the following: intention (individuals who engage in non suicidal self-injury do not desire to end their life), lethality and frequency (high letality methods with low frequency in suicidal behaviors). Attempted suicide affects 4-10 % of the case-subjects aged less than 15 years, and 54 % of the case-subjects under 24 years [21]. In the years of adolescence attempted suicide is approximately twice as frequent in females than in males; moreover 30 % of the subjects who have attempted suicide during adolescence will try again within 4 years [21]. Other studies report that 42 % of the adolescents who have attempted suicide will pursue it again within 2 years [22]. The repetition of an attempted suicide appears to be associated mainly with psychiatric disorders, particularly major depression, and difficulty in adjusting the emotions. Although several studies on repeated attempted suicide have been conducted, there is little data on prognostic factors that can predict the risk of recurrence. A study on adolescents with a history of single or multiple attempted suicides reports that subjects who have tried several times to commit suicide often show a related desire to die in their attempts, and regret to have not been able to complete their act; however, in this study, the specific characteristics of the attempted suicide that can predict a future attempt are not analyzed [23]. In particular it would be useful to understand if any change or escalation in the methods used to attempt suicide could provide any information to establish the likelihood of completing the act. A study on 1397 adults who completed suicide showed that the methods they had used in the previous attempts of suicide were less lethal [24]. A recent study [25] on 54 adolescents who attempted suicide has analyzed prognostic factors for recurrence of the act; 33 % of the subjects repeated suicide and the more indicative prognostic factors were: living alone, expressing a strong and real desire of dying, planning the act for an hour or more. When a child or an adolescent is admitted to the Emergency Room after having attempted suicide, the pediatrician must be able to assess the risk of repetition of this act. The subjects whose characteristics are listed in Table 4 may be recognized at an increased risk for recurrence and should always be hospitalized and subjected to a psychiatric evaluation [21].

**Table 4:** Risk Factors for recurrence of attempted suicide

Case history:	Demographic data:
- Constant thoughts of suicide	- Living alone
- Previous attempted suicide	- Male gender
Mental state:	
- Depression, mania, hypomania, anxiety, or all of these mental states	
- Substance abuse	
- Irritability, agitation, hallucinations, violent attitude	

Adapated from: Shaffer D, Pfeffer CR et al. Practice parameter for assessment and treatment of children and adolescents with suicidal behavior. J Am Acad Child Adolesc Psychiatry. 2001; 40(7 Supplement): 24S-51S)
  Before discharging the patient, the pediatrician should always recommend parents to remove weapons or potentially lethal drugs from home and to limit the access to alcohol or other substances with disinhibitory effects as much as possible. In addition, it is very important to make sure that the child/adolescent has a figure of support at home, and to ensure the family plan an adequate follow-up [21].
- Family factors. Familiarity for suicidal behaviors is a risk factor for suicide regardless of the presence of psychiatric illnesses. For this reason, the pediatrician should investigate and take into consideration any strife in the family, loss of a family member (divorce/death), possible abuse or relationship difficulties between parents and children [21]. In particular, a recent review [26] showed a close association between suicidal behaviors and a parent/child relationship characterized by poor care or overprotection.
- Substance abuse. Abuse of drugs and alcohol should always be investigated, since it increases the possibility of suicide, especially among male adolescents with humor disorders [27]. A recent study [28] examined the correlation between attempted suicide and “Heavy Episodic Drinking” (HED), a phenomenon increasingly observed among young people: HED increases the risk of suicide by 2.6 times in children under 13 years and by 1.2 times in subjects aged more than 18 years. Moreover, another study showed that drinking alcohol when one feels “low” increases the risk of attempted suicide by 3 times [29].
- Sexual and physical abuse. Sexual and physical abuse are important risk factors for suicidal behavior or suicidal ideation, especially in subjects aged between 16 and 25 years [21]. A recent study [30] showed that 50 % of women and 33 % of men who attempted suicide experienced physical or sexual abuse or domestic violence. Interventions to reduce the incidence of abuse or violence in pediatric age may represent a form of suicide prevention in adulthood [30].
- Sexual orientation. The pediatrician should also assess the sexual orientation of adolescents who are at risk of suicide. Literature shows that young homosexual men are 2-3 times more likely to commit suicide compared to their heterosexual peers [31]. In particular, those living in families that refuse their sexual orientation have a risk of suicide 8 times greater than those whose sexual orientation is accepted by the family [32].
- Bullying. It was reported that both “bullies” and “victims” of bullying are at an increased risk of suicidal behaviors [21]. In fact “bullies” are naturally more exposed to legal issues (criminal behaviors, including the use of drugs), and are at risk of developing an antisocial personality disorder. Being the victim of bullying affects mental growth in terms of self-esteem and personal well-being, and may lead to the development of depressive syndromes or of anorexia nervosa in predisposed individuals [33].
- Finally, safety of the home environment should also be taken into consideration [12]; environmental precautions are aimed at restricting access to means of suicide (eg guns, ropes, medications), and family members should be aware of the risks related to the situation [34]. In particular literature supports the importance of education in injury prevention: Kruesi [35] showed that educating parents in this field in the emergency room, leads to actions such as limiting the access to lethal means, for example locking up their firearms. On the other hand Brent [36] demonstrated that parents of depressed adolescents are frequently noncompliant with recommendations to remove firearms from the home. Restriction of access to means for suicide is a fundamental suicide prevention strategy in adolescents. Limiting availability of firearms in countries with high prevalence of suicide by shooting is mandatory: presence of firearms in households increases the risk of adolescent suicide and restriction of their availability reduces this risk [37]. Limiting availability of storage of pesticides in rural areas of developing countries, where suicide by pesticide ingestion is common, has also been suggested [38] In addition limiting the availability of pack sizes of drugs that are commonly used for self-poisoning in young people, such as paracetamol, is imperative [39].

Along with risk factors, pediatricians should also investigate also the presence of protective factors (Table 5) [31].

**Table 5:** Protective factors against suicide at a young age

- Access to services of mental hygiene
- Positive connections with the school
- Family stability
- Religious involvement
- Lack of access to deadly weapons
- Recognition of the importance of behavior intended to research of the aid of adults
- Good relations with their peers
- The ability to solve problems and overcome adversity

Thereafter, if suicidal ideation is suspected, the following specific questions should be investigated:

1. Have you ever thought to kill yourself or would you rather be dead?
2. Have you ever done something with the purpose to do yourself harm or kill yourself?
3. Have you ever considered methods to put an end to your existence?

Although direct questions can cause anxiety for the doctor, it is important to know that investigations about suicidal ideation do not induce such ideas in the patient [40]. Instead, it is of vital importance to understand the real need for immediate action [41]. The pediatrician must consider warning signs as they can provide information about the current psychophysical state of the patient. Warning signs must be differentiated from the risk factors and what best differentiates them is the temporal relationship with the risk of suicide. Alarm signals help in identifying subjects who may attempt suicide in subsequent minutes, hours or days, while risk factors identify the possibility of suicide in the long term (weeks or years) [41]. Over the years, several attempts have been made to define these alarm signals; in 2006 the American Association of Suicidology [42] published a Consensus that can be applied in clinical practice: the sentence “IS PATH WARM” is useful to recall the main “warning signs” (Table 6). It is also important to differentiate the need for immediate assistance (Table 7), not only for the pediatrician, but also for educators, teachers, family, and the same adolescents.

**Table 6:** Consensus warning signs of suicide

A person at risk for suicidal behavior most often will exhibit warning signs such as:		
I	Ideation	Expressed or communicated ideation
		Threatening to hurt or kill him/herself or talking of wanting to hurt or kill him/herself
		Looking for ways to kill him/herself by seeking access to firearms, available pills or other means
		Talking or writing about death, dying or suicide when these actions are out of the ordinary
S	Substance abuse	Increased substance (alcohol or drug) use
P	Purposelessness	No reasons for living; no sense of purpose in life
A	Anxiety	Anxiety, agitation, unable to sleep or sleeping all the time
T	Trapped	Feeling trapped – like there’s no way out
H	Hopelessness	Hopelessness
W	Withdrawal	Withdrawing from friends, family and society
A	Anger	Rage, uncontrolled anger, seeking revenge
R	Recklessness	Acting reckless or engaging in risky activities, seemingly without thinking
M	Mood changes	Dramatic mood changes

Adapted from: American Association of Suicidology. How do you remember the warning signs of suicide? Newslink 2006; 32:20

**Table 7:** Consensus warning signs of suicide

Seek help immediately when you see one of these behaviors:
- Someone who threatens to kill or hurt themselves
- Someone means to kill themselves: medicines, weapons and other means
- Someone who speaks or writes about death or suicide
Seek help, without the need of immediate assistance, when you see someone that manifests itself:
- Complete loss of hope
- Anger, rage, a desire for revenge
- Imprudence and desire to carry out dangerous activities
- Feeling of being trapped, no way out
- Abuse of alcohol/drugs
- Isolation from friends, from family or from society
- Anxiety, agitation, insomnia or hypersomnia
- Excessive change in tone of the mood
- Absence of a reason to live, absence of purposes in life

Adapted from: American Association of Suicidology. How do you remember the warning signs of suicide? Newslink 2006; 32:20

Finally, a very debated theme is the use of antidepressant drugs in pediatric age, literature and long-term data for this is lacking. After a post hoc analysis that showed a significant increase in suicidal behaviors in patients treated with paroxetine, the FDA conducted a meta-analysis [43] in which data of 24 clinical trials in 4.400 pediatric patients suffering from major depression, obsessive compulsive disorder and other psychiatric disorders treated with antidepressants were analyzed. The results showed an increase in suicidal ideation during the first months of therapy in patients treated with antidepressants respect to placebo (4 % versus 2 %) but no suicide brought to term. On 15th October 2004 [44], the Food and Drug Administration ordered all the pharmaceutical companies to add a warning black box on all packages of antidepressants recommending the following: “All pediatric patients following a therapy with antidepressants for any disease should be closely monitored by evaluating a possible worsening of symptoms/signs of suicidal behaviors or any changes in behavior, in particular in the first months of therapy, or if they are implementing changes of dosage. Ideally, the patient should be evaluated once a week for the first 4 weeks, then every other week for the next 4 weeks, and then 12 weeks after the initiation of therapy. If clinically indicated the patient should also be evaluated after 12 weeks. Furthermore, in the time interval between visits, it would be advisable to contact the patient by phone in order to get information about his state of health”. Retrospectively, some Authors [45] showed that the analysis conducted by the FDA had some limitations: firstly, adverse effects were only studied for a short period of time, secondly, drugs were not assessed individually, and thirdly, not all the methods used to assess the risk of suicide had been approved for pediatric patients. A recent review [45] has therefore analyzed the consequences of the introduction of the “black box” in the packages of antidepressants. The first noticeable result, starting from 2005, was the reduction in the number of subjects diagnosed with depression in pediatric age both in the USA and in Europe. Secondly, up to 2004, the prescription of antidepressant drugs in the pediatric age had presented a significant increase, both in the United States and in some countries in Europe; however from 2005 onwards, the number of prescriptions has shown a significant reduction, especially among pediatricians, doctors of general medicine and, to a lesser extent, psychiatrists; the review also showed that a high percentage of parents refused antidepressant treatment for their siblings [46]. Studies have also shown that, after the introduction of the “black box”, doctors pay greater attention in monitoring patients on antidepressants, especially in the first weeks of therapy [47]. In addition, some Authors [48] reported an increase in the number of suicides among children and adolescents in the period post “black box”, assuming then an association with the reduction of prescription of antidepressant drugs. This issue still remains highly controversial and long-term studies are needed to fully evaluate the efficacy and safety of antidepressant drugs in pediatric age and their possible association with suicidal events. However, the American Academy of Child and Adolescent Psychiatry and the American Psychiatric Association [49] recommend a careful monitoring of pediatric patients even at home, by encouraging parents to contact their physician in the event of an outbreak of the signs/symptoms summarized in Table 8.

**Table 8:** Alarm signals whilst on antidepressant therapy

- Frequent thoughts or wish of death
- Self-destructive behaviors
- Signs of excessive anxiety/panic, agitation, aggression, impulsivity, insomnia, and irritability
- Onset or worsening of signs of restlessness
- Excessive euphoria or energy
- Rapid speech
- Setting goals too difficult to reach

Adapted from: American Psychiatric Association, 2001

## Conclusions

The data reported highlights the fundamental role of the pediatrician in the management of the child/adolescent with suicidal behavior. He/she should be able to investigate the presence of mood disorders, suicidal thoughts, sexual orientation and other factors related to suicide, and educate those who live and work with young people (for example parents and teachers) to recognize the risk factors for suicide, with a particular focus on patients with chronic illnesses. In addition, the pediatrician should be able to collaborate with other professionals (e.g., psychiatrists) involved in the care of patients at risk for suicide or attempted suicide, should be aware of the balance between benefits and risks of administering antidepressant drugs, and should monitor the patients whilst on treatment, in particular when starting antidepressant and after any dose-changing.
